# A search and rescue robot search method based on flower pollination algorithm and Q-learning fusion algorithm

**DOI:** 10.1371/journal.pone.0283751

**Published:** 2023-03-30

**Authors:** Bing Hao, Jianshuo Zhao, He Du, Qi Wang, Qi Yuan, Shuo Zhao

**Affiliations:** 1 College of Computer and Control Engineering, Qiqihar University, Qiqihar, China; 2 College of Telecommunication and Electronic Engineering, Qiqihar University, Qiqihar, China; Zonguldak Bülent Ecevit University: Zonguldak Bulent Ecevit Universitesi, TURKEY

## Abstract

Search algorithm plays an important role in the motion planning of the robot, it determines whether the mobile robot complete the task. To solve the search task in complex environments, a fusion algorithm based on the Flower Pollination algorithm and Q-learning is proposed. To improve the accuracy, an improved grid map is used in the section of environment modeling to change the original static grid to a combination of static and dynamic grids. Secondly, a combination of Q-learning and Flower Pollination algorithm is used to complete the initialization of the Q-table and accelerate the efficiency of the search and rescue robot path search. A combination of static and dynamic reward function is proposed for the different situations encountered by the search and rescue robot during the search process, as a way to allow the search and rescue robot to get better different feedback results in each specific situation. The experiments are divided into two parts: typical and improved grid map path planning. Experiments show that the improved grid map can increase the success rate and the FIQL can be used by the search and rescue robot to accomplish the task in a complex environment. Compared with other algorithms, FIQL can reduce the number of iterations, improve the adaptability of the search and rescue robot to complex environments, and have the advantages of short convergence time and small computational effort.

## Introduction

With the rapid development of artificial intelligence and sensors, search and rescue robots (SAR robots) are no longer far away, and SAR robots can help humans to complete search and rescue tasks, especially in the era of prevalent epidemics and frequent natural disasters. As technology advances, the functions, movements, and responses of SAR robots are becoming more and more mature, bringing hope to people in deep water. Compared to real humans, SAR robots can not only detect the target that needed to be searched and rescued but can also do things that humans cannot do, such as snake-like robots that can complete the search task in narrow environments. At the same time, the SAR robot also has a human that does not have the "superpower": taking pictures, navigation, positioning, changing the structure, and other functions. SAR robots have a wide range of search and rescue and can be applied to search and rescue missions in waters and airspace [1], in addition to being able to conduct search and rescue on land [2] and in ruins [3]. SAR robots do not only involve several disciplines such as automation technology and artificial intelligence, but also involve multi-domain knowledge such as instant localization [4, 5], sensor information fusion [6], and path planning [7]. Robot path planning techniques have been applied in various areas of human production and life. SAR robot path search is the need for SAR robot to plan a safe, collision-free path from the initial position to the target position in different work scenarios. For example, in natural disaster rescue, Unmanned Aerial Vehicle (UAV) formations are able to avoid obstacles to plan a better set of paths to reach a given region [8]; In the military domain, UAVs and robots are required to plan a better path to complete the mission [9]; In daily life, food delivery and courier logistics robots need to plan an optimal path to meet the needs of people [10]. And human-robot cooperate with each other to complete specific tasks [11, 12].

Many algorithms enable target search for SAR robots, and these can be broadly classified as traditional algorithms, intelligent algorithms and reinforcement learning algorithms. Traditional algorithms includes:A* [13], Artificial potential field(APF) [14]. The basic idea of APF is the target point exerting an "attractive force" on the agent and the obstacle exerting a "repulsive force" on the agent. But this algorithm have local optimal point problem. The A* algorithm is a common heuristic path-finding algorithm, which was published by Peter Hart et al. in 1968. The A* algorithm can control the speed and accuracy of the algorithm by adjusting the heuristic function. However, as the number of data increases and the number of useless nodes increases, the search time of the A* algorithm grows and thus the convergence speed of the algorithm decreases. Intelligent algorithms includes:Genetic Algorithm(GA) [15], Ant colony optimization(ACO) [16] and Particle swarm algorithm. To solve the problem that the single robot task execution capability is not enough, literature [17] proposed a multi-robot path planning and formation cooperative control to complete the task. Firstly, an improved global path planning model based on a particle swarm optimization algorithm is proposed to solve the problem of low efficiency of robot path planning. Secondly, the simulated annealing algorithm is introduced to optimize the traditional artificial potential field method. The results show that the improved algorithm improves the exploration ability of robot formation, effectively avoids obstacles. The GA algorithm formed by simulating the theoretical basis of natural selection, heredity, and variation in biological evolution. The advantage of the GA is that it can handle multiple individuals at the same time and cover a large area, so it is more suitable for solving nonlinear problems that are difficult to solve by traditional search methods. The ACO is designed to simulate the foraging activities of ants, which leave a certain of pheromones on their foraging paths. The shorter path, the higher concentration of pheromones, so the more ants choose this path, and the more pheromones are left behind, the higher chance of being selected. ACO not only has the global search ability of the population but also has the cooperation among individuals. Even if it does not know the complete information about the environment, it can find a better path. However, in the early stage of the algorithm, the convergence speed is slow, which requires a lot of calculation time. Reinforcement learning [18] is one of the artificial intelligence algorithms that learn to achieve goals in uncertain, potentially complex environments by training a machine learning model to make a series of decisions that enable the robot to choose the actions to perform based on the environment. Reinforcement learning was widely used in different fields. The author in [19] proposed a new robust hybrid classification model based on swarm optimization-supported feature selection to classify diseases in apple, grape, and tomato plants with high accuracy in real time. The disease classification model was consist of FPA, support vector machine (SVM) and convolutional neural network (CNN). The experimental results show that the proposed model classifies the specified plant leaf diseases in real time with high accuracy. To take advantage of historical data to optimize maintenance policies for multi-component systems, paper [20] proposed an artificial-intelligence-based maintenance approach. The proposed approach constructs a predictor based on an artificial neural network (ANN) for estimating maintenance cost at a system level and uses a deep reinforcement learning algorithm to optimize maintenance decisions.

Q-learning is a reinforcement learning algorithm that allows robots to learn like humans. Robots in unknown environments gradually acquire information about the environment by continuously interacting with it. In this process, the robot gets a positive reward value if it does what we expect it to do, and we punish the robot if it does something we don’t allow. After repeated iterations, the robot can then fully understand the mapping relationship from states to actions. The robot can then search for the optimal path by maximizing the cumulative payoff. However, Q-learning algorithms have limitations: slow convergence and exploration-utilization dilemma problems [21]. To speed up the convergence, an improved Q-learning (IEGQL) algorithm is proposed in the literature [22] to address the shortcomings of the slow convergence of the traditional Q-learning algorithm to improve the efficiency in terms of path length and computational cost, in addition to a new mathematical model that provides optimal choices while ensuring fast convergence. A path planning algorithm based on deep Q-learning and Convolutional Neural Networks (CNN) algorithm was proposed in the literature [23], and simulation results showed that the algorithm can achieve flexible and efficient robot motion and can reduce the convergence time of the robot in various environments compared to traditional methods. The literature [24] presents a reward matrix-based Q-learning algorithm that meets the path planning needs of marine robots.

Although the above algorithm can reduce the search time of Q-learning and improve the search efficiency, but the modeling of robots in complex environments is not considered in detail. In this paper we have improve the accurate of the environment modeling. In addition, the reward function of the improved algorithm can be more reasonable based on the feedback of the SAR robot.

Using Q-learning for path planning is that the SAR robot can learn independently in a complex environment, and can increase the applicability of the algorithm by combining it with the neural network. In addition, the evaluation strategy of Q-learning is relatively simple. To solve the search problem of SAR robots in complex environments, this paper proposes a fusion algorithm (FIQL) based on the Flower Pollination algorithm (FPA) and an improved Q-learning algorithm. This Fusion algorithm combined static and dynamic reward functions considered the accurate distribution of obstacles and used FPA to complete the initialization of the Q-table to accelerate the efficiency of algorithm initialization. The main key contributions of this paper are as follows:

1. To address the drawback of the low accuracy of the conventional grid map [25] for obstacle modeling, an improved grid map is used in the environment modeling section to change the original static grid to a combination of static and dynamic grids to increase the accuracy of the SAR robot for obstacles around the search target.
2. The combination of FPA and Q-learning algorithm, using FPA to initialize the Q-table, reduces the computational effort of the algorithm, speeds up the convergence of the algorithm, and precisely classifies the obstacles close to the search target using an improved grid map construction method.
3. The reward function of the improved Q-learning algorithm influences the action selection of the SAR robot at all times by setting static and dynamic reward values. When the SAR robot completes target tracking or collides with an obstacle, it receives a static reward value. In the rest of the cases, the SAR robot will receive dynamic reward values to motivate it to choose the appropriate action according to its specific situation.
4. By comparing the algorithm of proposed FIQL with other algorithms, the result shows that the improved map can improve the success rate of the algorithm, and the improved algorithm has better performance than the other algorithms.

## Materials and methods

### Problem description

**Fig 1:**
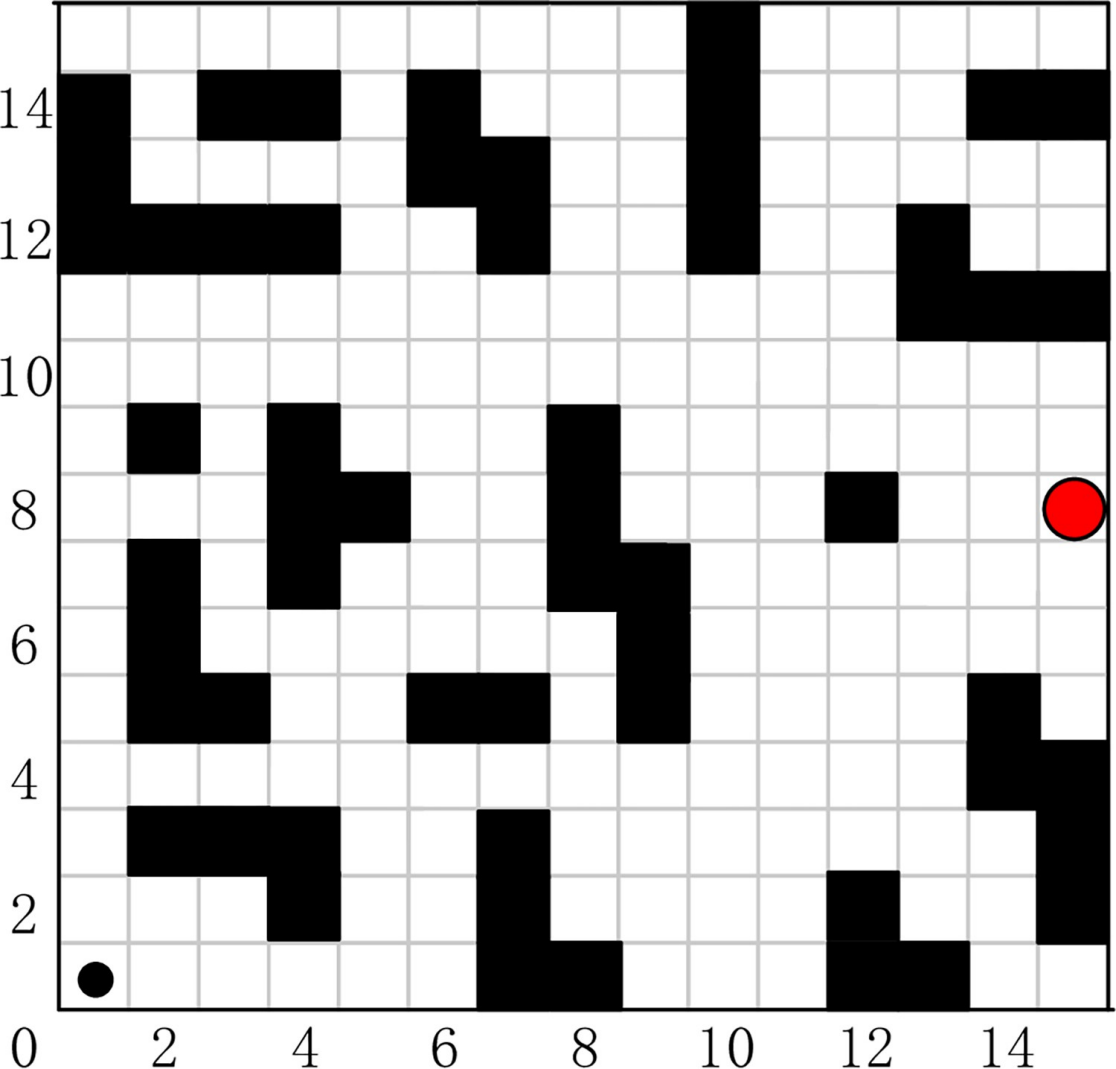
SAR robot movement space.

To understand the target search behavior of the SAR robot, we can imagine the task of a real-life SAR robot: the SAR robot has to achieve a safe, collision-free, and close enough path for the SAR robot to successfully reach the search target location based on the location of the search target and the location of the surrounding obstacles. The searching and rescuing scenarios of SAR robot are different, and the location of the SAR robot search target is not fixed. The SAR robot needs to know the location of the search target and the distribution of obstacles around it at all times. For convenience, we define SAR robot and search targets as circles of different sizes, and the starting positions of SAR robot and their search targets may not be identical. The motion space of the SAR robot as shown in Fig 1, where the white grid represents the feasible area; the black grid represents the obstacle; the black circular object and the red circular object represent the SAR robot and its search target.

### Reinforcement learning

Reinforcement learning is the process by which a robot learns how to map environmental states to its own behavioral actions in order to obtain the maximum reward value, in which the robot needs to look for actions with larger rewards, avoid actions with low rewards, or get penalized. The problem of reinforcement learning can be simplified arithmetically using the Markov decision process (MDP) [26]. The goal of reinforcement learning is to find a strategy to maximize the expectation of future rewards $E(\sum_{k=0}^{\infty }\gamma^{k}R_{t+k+1})$, where *R*_*t*+*k*+1_ is the reward at the next moment and *γ* is the discount factor. Reinforcement learning can be expressed in terms of a quintet: RL=〈S,A,P,R,γ〉.

**Fig 2:**
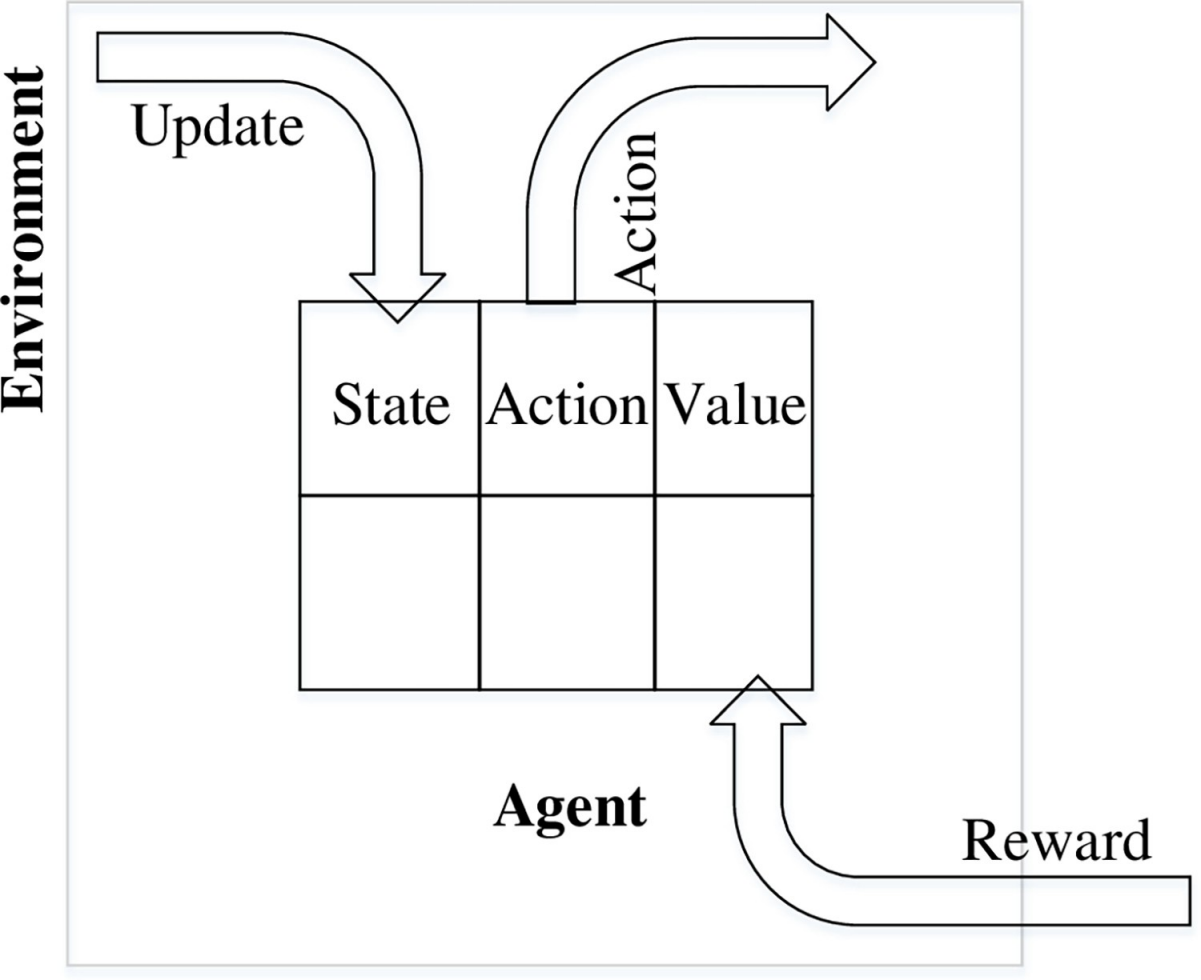
Interaction models for reinforcement learning.

Where, *S* denotes the state set (states), *A* denotes a set of actions (actions), *P* denotes the state transfer probability, *R* is the payoff function, and *γ* is the discount factor. Fig 2 depicts a basic process for the robot to interact with the environment.

Such methods are said to be model-independent [27] (Model-free) if the robot does not need to know the full content of the quintuplet: RL=〈S,A,P,R,γ〉 during the learning process. If the learning process starts with learning the model knowledge of the quintet and then deriving and learning the optimal strategy based on these, we call it model-related [28] (Model-based). Mobile robots sometimes do not need to model the environment to find optimal strategies, and one of the most classic examples is Q-learning [29]. The Q-learning algorithm is a classical reinforcement learning algorithm. The literature [30] demonstrated that Q-learning can be used to solve multi-robot path planning problems by first pre-training the Q-learning algorithm to make it suitable for path planning. Then a map model of the obstacle environment is built and a path planning program is prepared by applying the state action-value function. Finally, the experimental results are collected, and the experimental results show that the Q-learning algorithm can successfully solve the multi-path planning problem. The literature [31] gives the design process and demonstrates the feasibility of building a kinematic model of a quadrotor UAV, abstracting the environment in the form of a grid world, and using Q-learning algorithms. Q-learning uses five tuples to describe the path planning problem. One of the Q-learning updates is as follows:

$$Q(S_{t},A_{t})=Q(S_{t},A_{t})+\alpha (R+\gamma Q(S_{t+1},A_{t+1})-Q(S_{t},A_{t}))$$ (1)

where, *α* represents the size of the robot’s learning rate, when *α*→0 means the robot tends to use more current information; when *α*→1 means the robot tends to consider more the impact of future actions. And *γ* is the discount factor, which represents the importance of future rewards. *S*_*t*_, *S*_*t*+1_ is the current state and next state of SAR robot. The robot determines the next action by selecting the optimal Q-value in the Q-table with the following expression:

$$A^{*}(S)=\max Q(S_{t+1},A)$$ (2)

### FIQL algorithm

In this section we elaborate on the proposed FIQL algorithm for solving the search problem of the SAR robot, before that, it is necessary to understand the starting and target point coordinates of the SAR robot, and the environment modeling is mainly to accurately represent the working environment information in a more convenient and simplified way. Up to now, there are more mature methods applicable to environmental map construction, and the commonly used map construction methods can be roughly divided into grid map method, visual map method [32], Voronoi [33] map method, etc. To better describe the studied content, SAR robot has the following premises:

1. SAR robot path planning studied in this paper is divided into two parts:typical and improved grid map path planning. The location, shape and size of obstacles in the environment are known in the path planning.
2. SAR robot and obstacles around it are three-dimensional objects in practice. This paper treats their motion space as two-dimensional coordinates to simplify the problem.

### Typical grid map

**Fig 3:**
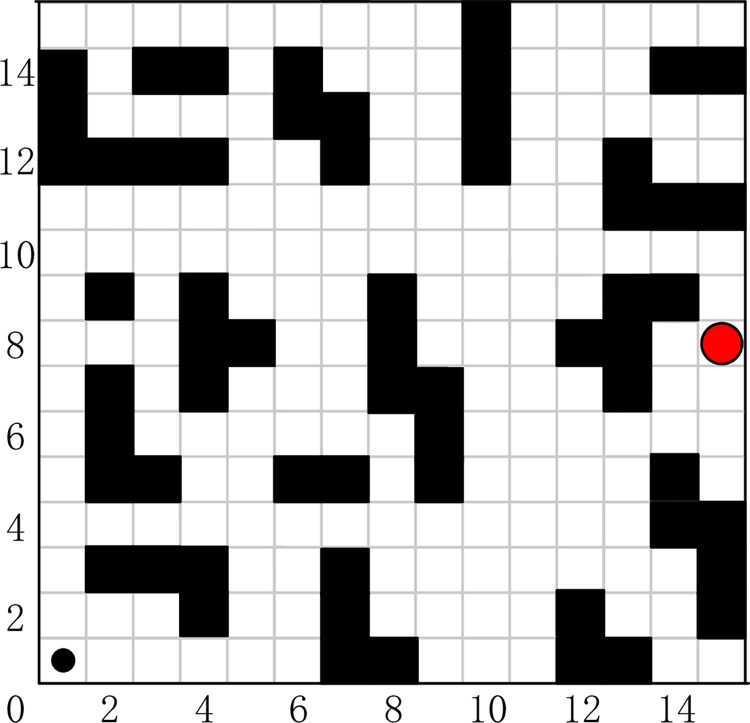
The classical grid map.

The grid map method originated from in solving the traveler problem, and it is one of the more widely used methods for solving path planning problems today. The grid map is a depiction of the working environment in which the robot operates by means of binary cell grid information. The environmental information is gridized to obtain a grid map, where the grid map occupied by the obstacle is called the obstacle grid and is represented by the value 1. Conversely, the rest of the grid map is said to be a free grid and is represented by the value 0. The traditional grid map uses the same way for obstacles of different sizes, obstacles that do not occupy a grid size are also treated according to a grid size, which leads to the inaccuracy of map modeling and brings trouble to the subsequent search of SAR robot. The grid map before improvement is shown in Fig 3. When the grid map method is used to partition the environmental space, the size of the grid map needs to be determined based on the robot form factor size. In the literature [34], an IDC-FM2 method-based fast global path planning algorithm for two-level SR grid maps is proposed for unmanned surface vehicle missions with short time requirements in large-scale complex environments, which is able to obtain continuous, smooth, and quasi-time-optimal paths when bypassing obstacles while maintaining a safe distance around them. As the path approaches the obstacle, it is restricted to a safety region determined by two nearshore distance parameters. By adjusting these two nearshore distance parameters, the path can be flexibly modified.

#### Improved grid map

**Fig 4:**
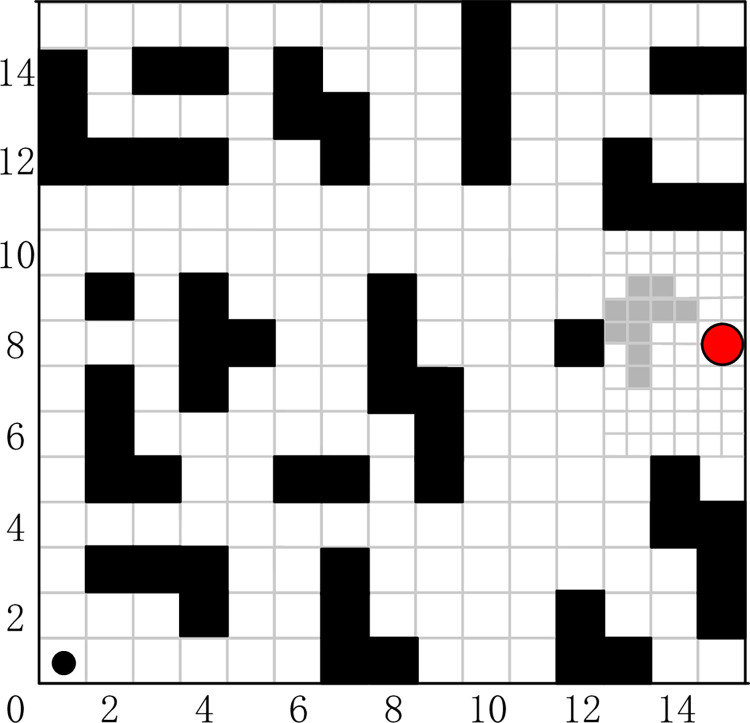
The improved grid map.

An improved grid map is proposed to improve the disadvantages of the traditional grid for the inaccurate modeling of obstacle environment, and the grid map is divided into static grids and dynamic censored grids. For the design of the obstacle, we use the external square of the external circle of the obstacle for the design of the obstacle to replace the size of the actual obstacle. The expansion process can provide a safety threshold for the SAR robot and reduce the possibility of collision between the SAR robot and the obstacle. The grid map before and after the improvement is shown in Figs 3 and 4.

#### Static grid

The static grid is set up in the same way as the traditional grid map, the SAR robot is defined as a circle with radius *γ*, the SAR robot search target is defined as a circle with radial length *R*, and the coordinates of the center of gravity locations of the SAR robot and the SAR robot search target are (*x*_*d*_, *y*_*d*_) and (*X*_*p*_, *Y*_*p*_), respectively. The lateral and vertical distances between the center of gravity positions between the SAR robot and the SAR robot search target are $L=\|X_{p}-x_{d}\|$ and $D=\|Y_{p}-y_{d}\|$. Define a rectangle of size L*D for the active environment searched by the search and rescue robot, and divide the active environment with two sides of length *L* and *D* into m*n square static grids of *m* rows and *n* columns with side length *d*_1_. The value of each grid in a static grid map is also represented by 0 or 1.

Dynamic grid. In order to reach the target point more accurately and reduce the search path length, the dynamic grid is quadratically divided according to the size of the static grid to improve the accuracy of the modeling. In a traditional grid map, if an irregular obstacle occupies only a quarter of a grid, then it is also calculated according to grid size, if an irregular obstacle occupies only a quarter of a dynamic grid, then it is modeled according to the actual size and does not need to be calculated according to a grid size uniformly. The improved grid map is shown in Fig 4, where the gray grid is a more accurate representation of the obstacle based on the dynamic grid.

At time *t*, the center of gravity $\left(X{\left(t\right)}_{p},Y{\left(t\right)}_{p}\right)$ of the SAR robot search target is the center point, and a square range with side length *H* is selected, where $H=(V_{p}(t)/d_{2})*R$ and *V*_*p*_(*t*) is the current movement speed of the SAR robot search target. The dynamic square range is divided into s*s square random grids with side length *d*_2_, and the grids less than one are rounded upward.

where, *d*_1_ = 4*r*, *d*_2_ = 2*r*, *m* = ⌈*L*/*d*_1_⌉, *n* = ⌈*D*/*d*_1_⌉, *s* = ⌈*L*/*d*_2_⌉;

#### Action selection

In order to achieve the SAR robot that can complete the target search task in as little time as possible and reduce the data storage of the Q-table. Taking into account the size of the SAR robot, it is assumed that it can reach eight adjacent grids, and for this purpose, eight movement directions (Top, Top right, Right, Bottom right, Bottom, Bottom left, Left, and Top left) and corresponding movement distances are defined. $A=\{a_{1},a_{2},a_{3},a_{4},a_{5},a_{6},a_{7},a_{8}\}$ represents the motion space of the SAR robot:

action1: $a_{1}=\mathrm{move}\;\mathrm{up}\;dm;$

action2: $a_{2}=\mathrm{move}\;\mathrm{up}\;\mathrm{to}\;\mathrm{the}\;\mathrm{right}\;\sqrt{2}dm;$

action3: $a_{3}=\mathrm{move}\;\mathrm{right}\;dm;$

action4: $a_{4}=\mathrm{move}\;\mathrm{down}\;\mathrm{to}\;\mathrm{the}\;\mathrm{right}\;\sqrt{2}dm;$

action5: $a_{5}=\mathrm{move}\;\mathrm{down}\;dm;$

action6: $a_{6}=\mathrm{move}\;\mathrm{down}\;\mathrm{to}\;\mathrm{the}\;\mathrm{left}\;\sqrt{2}dm;$

action7: $a_{7}=\mathrm{move}\;\mathrm{left}\;dm;$

action8: $a_{8}=\mathrm{move}\;\mathrm{up}\;\mathrm{to}\;\mathrm{the}\;\mathrm{left}\;\sqrt{2}dm;$

where, the values are determined by the type of grid the SAR robot is currently in. When the search and rescue robot is in a dynamic grid *d* = *d*_2_, else *d* = *d*_1_.

#### Reward functions

The reward function is used to judge the value of the behavior, and the SAR robot interacts with the environment according to the reward function and adjusts the selection action strategy by the reward value, and the appropriate reward function helps to reinforce the desired behavior and punish the improper behavior. In previous reinforcement learning path planning, the reward value is usually a static constant [35], which will lead to a blind random search in the environment by the SAR robot and increase the convergence time. The literature [36] combines heuristic search strategies, simulated annealing mechanisms, and reactive navigation principles to Q-learning-based Dyna architecture, a novel action selection strategy is proposed that, together with heuristic reward functions and heuristic actions, can solve the exploration-utilization dilemma and improve global search performance, convergence, and learning efficiency. We propose a combined static and dynamic reward function setting in the FIQL algorithm, which provides the target point and the current position as a priori knowledge to the search and rescue robot, and the larger the reward obtained when it is closer to the target point, prompting it to move in the direction of the target point and accelerating the convergence speed.


$$R_{t=}\{\begin{array}{ll} 100 & \mathrm{Complete}\;\mathrm{the}\;\mathrm{target}\;\mathrm{search}\\ -100 & \mathrm{Collision}\;\mathrm{occurred}\\ -2Dis(S_{t}) & Dis(S_{t+1})\geq Dis(S_{t})\\ 2Dis(S_{t}) & Dis(S_{t+1})<Dis(S_{t})\\ -Dis(S_{t}) & \mathrm{else} \end{array}$$ (3)

Compared with the traditional reward function design, the reward function of the FIQL algorithm considers more situations: in addition to the SAR robot completing the search task, collision with obstacles to obtaining the reward value, in addition, *Dis*(*S*_*t*_) is the distance between the SAR robot in the current state and in the next state to the target point. If the distance between the search and rescue robot and the search target in state *S*_*t*_ is greater than the distance between the search target in the next state *S*_*t*+1_, it means that at this time the SAR robot moves in the direction of approaching the target point, at this time the SAR robot will get a positive reward value. On the contrary, If the distance between the search and rescue robot and the search target in state *S*_*t*_ is less than the distance between the search target in the next state *S*_*t*+1_, it means that at this time the SAR robot moves in the direction away from the movement target point, at this time the SAR robot will get a negative reward value. Finally, in order to better accelerate the convergence speed of the algorithm, the reward value for other cases is set to −*Dis*(*S*_*t*_).

#### Q-table initialization

**Fig 5:**
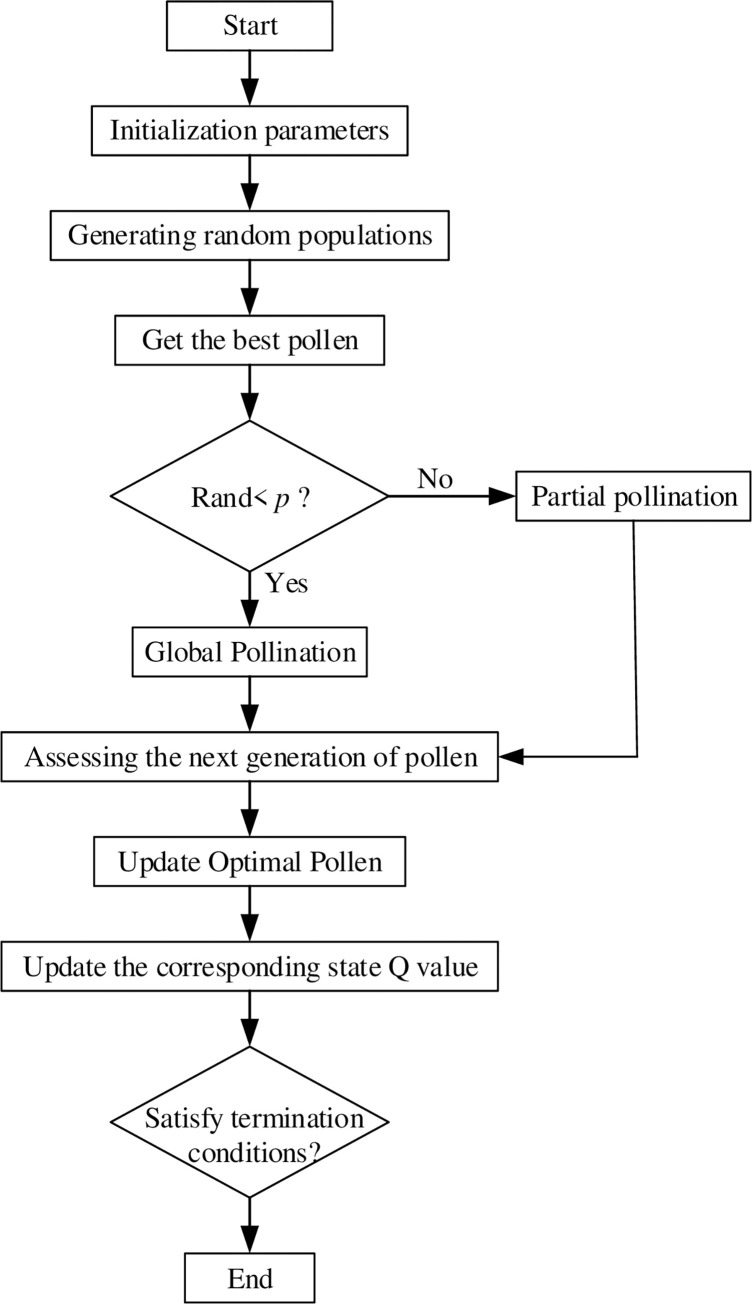
Initialize Q-table with FPA.

The Q-learning algorithm usually initializes the Q-table to 0 or a normally distributed random number without detailed consideration of the influence of the environmental prior knowledge guide on the SAR robot, resulting in the SAR robot only being able to randomly select actions during the exploration phase, causing slow convergence and long computation time. The FPA is a heuristic optimization algorithm, which is simulated and designed according to the mechanism of plant flower pollination behavior in nature. The local search and global search processes of FPA simulate the process of self-pollination and cross-pollination respectively, and balance the proportion distribution of the search process through random perturbation. The algorithm has the characteristics of simple structure, strong robustness, strong search ability and easy implementation. Therefore, FPA can be applied to optimization problems. To optimize slow convergence problem, this paper uses the FPA to initialization Q-table (Fig 5 is the specific flow chart).

The flower pollination algorithm simulates the flower pollination process of flowering plants in nature. During the global pollination process, the pollen position is updated by the formula:

$$X_{i}^{t+1}=X_{i}^{t}+L(X_{i}^{t}-g_{best})$$ (4)

where, *L* is the update step and the expression is as follows:

$$L∼\frac{\lambda \Gamma (\lambda )\sin(\frac{\lambda \pi }{2})}{\pi }\frac{1}{s^{1+\lambda }}$$ (5)

where $X_{i}^{t+1}$, $X_{i}^{t}$ are the solutions in the *t*+1 and *t* generations, respectively; *g*_*best*_ is the optimal solution in the current iteration; Γ(*λ*) is the standard gamma function.

The equation for updating the location of the local pollination stage is as follows:

$$X_{i}^{t+1}=X_{i}^{t}+\varepsilon_{1}(X_{j}^{t}-X_{k}^{t})$$ (6)

where, $X_{j}^{t}$ and $X_{k}^{t}$ are solutions different from $X_{j}^{t}$ and $X_{k}^{t}$ chosen randomly from within the population; *ε*_1_ represents the reproduction probability, which is a random number and *ε*_1_∈(0,1).


$$\left[\begin{array}{l} x_{1}y_{1}\\ x_{1}y_{2}\\ \vdots \vdots \\ x_{1}y_{D}\\ x_{2}y_{1}\\ \vdots \vdots \\ x_{L}y_{D} \end{array}\right]$$ (7)

To better initialize the pollen population *N*, each grid of the grid map with unit size *d*_1_ of L*D is first transformed into a two-dimensional matrix (Eq(7)), and each grid represents a different state, which is assigned to the corresponding grid state according to the free grid region, the grid where the search and rescue robot is located, and the grid where the search and rescue robot is searching for a target, as in 3,2,1, and other parameters are set in Table 1. Before initializing the Q-table using FPA, we need to determine the number of populations and then use Eq (2) to determine the location of the grid in which the highest state of Q-value is located, and then use global and local pollination based on the magnitude of a random number and the value of *p*, so as to continuously iterate and search for feasible paths. The optimal value obtained after each iteration is compared with *p*. If the optimal value is greater than *p*, the current position is the optimal position and it is updated to the Q-value of the current state. With this iteration, we can complete the initialization of the Q-table. After completing the initialization of the Q-table, a good foundation is laid for the SAR robot to complete the search.

**Table 1:** Parameter settings of FIQL, CQL, PSO, GWO and DA.

Algorithms	Parameter selection
FIQL	$\alpha =0.9-Iter*0.01,\gamma =0.9,N=8,P=0.8,\varepsilon_{1}=0.8,Max\_Iter=30$
CQL	α=0.9,β=0.9
PSO	$c_{1}=2,c_{2}=2$
	$\omega =\omega_{\max}-Iter*((\omega_{\max}-\omega_{\min})/100)$
	$\omega_{\max}=0.9,\omega_{\min}=0.2$
GWO	a=2−Iter*0.02,C=2*rand()
DA	a=2−Iter*0.02,C=2*rand()
	c=2*rand()*(0.1/0.1*Iter/50)
	e=0.1/0.1*Iter/50
	f=2*rand()
	r=3+(Iter/100)
	s=2*rand()*(0.1−0.1*Iter/50)
	$\omega =\omega_{\max}-Iter*((\omega_{\max}-\omega_{\min})/100)$
	$\omega_{\max}=0.9,\omega_{\min}=0.2$

**Fig 6:**
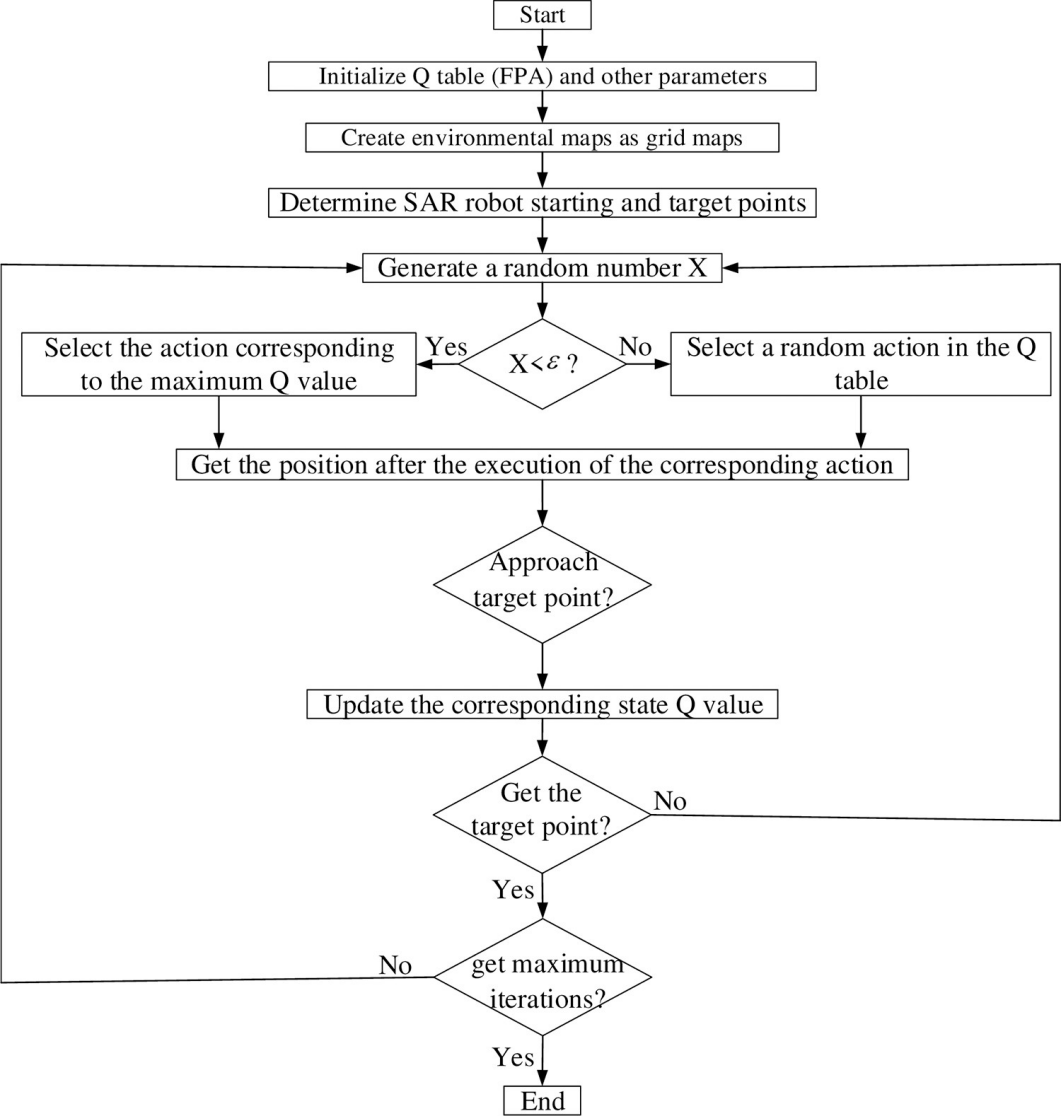
SAR robot search overall process.

After the initialization of the Q-table is completed, the improved grid map, Q-learning algorithm can be used to implement the search problem for targets by the SAR robot. Fig 6 describes the complete process of completing the search for targets by the SAR robot using FIQL.

Step1: The FPA algorithm is used to initialize the Q-table and initialize other parameters.

Step2: Determine the search environment for the SAR robot and model the actual environment as a static grid and a dynamic grid using an improved grid map.

Step3: Clarify the starting and ending points of SAR robot tracking.

Step4: By comparing the magnitude of the random number X with the exploration factor *ε*.

Step4.1: If the random number X is smaller, then the action corresponding to the maximum Q-value is selected.

Step4.2: If the random number X is larger, a random action is selected.

Step5: Determine the current position of the search and rescue robot.

Step5.1: If the SAR robot is close to the target, it is given a positive reward value to motivate it to get closer to the target.

Step5.2: If the SAR robot is far away from the target, it is given a negative reward value to make it perform a different action to approach the target.

Step6: Determine whether the termination condition is met, and if so, end the process.

## Results and discussion

### Simulation experiment environment and parameter setting

**Fig 7:**
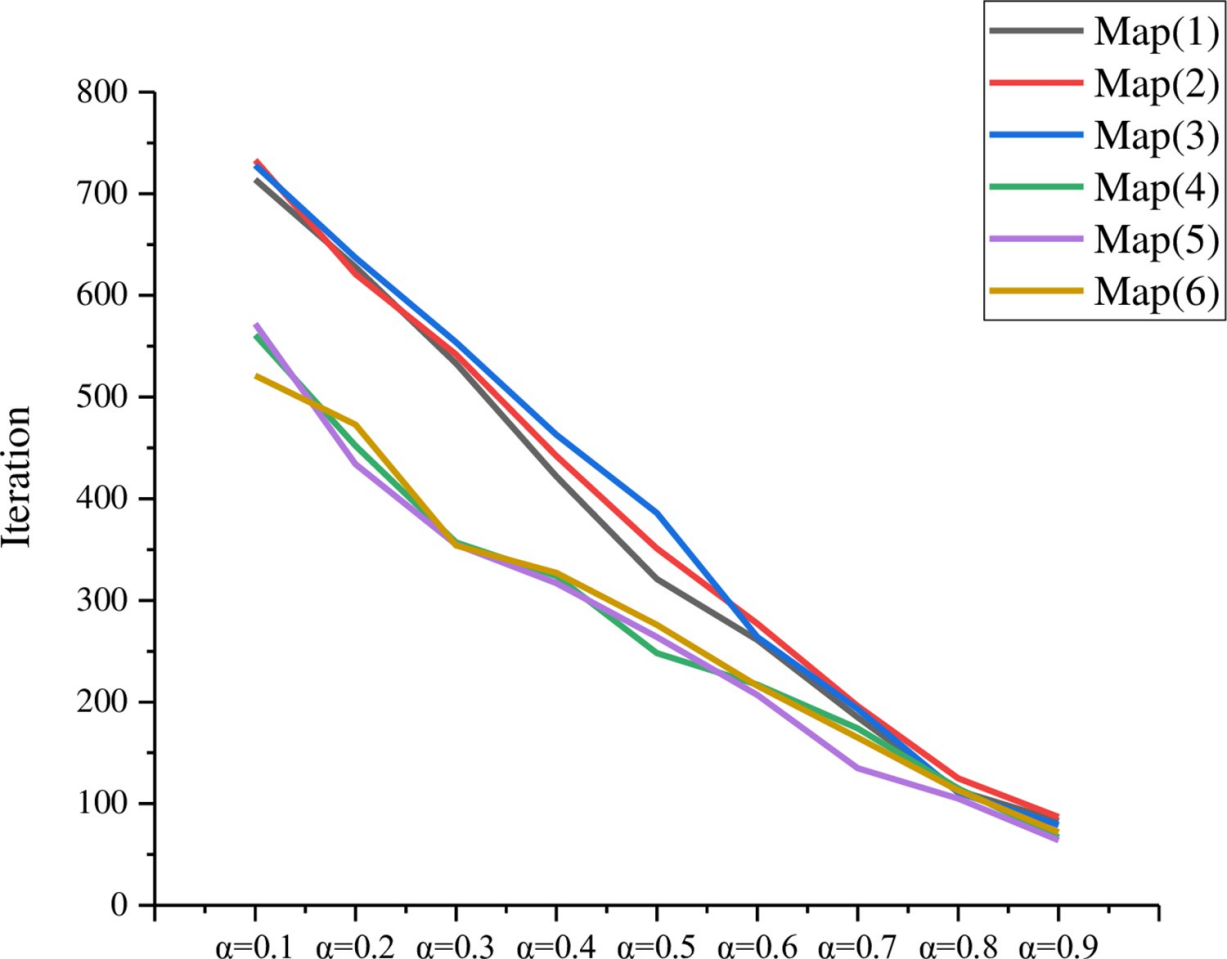
Iteration times with different learning factor.

In this paper, the simulation experiments are mainly conducted for the grid map environment constructed by six different maps, where the white grid represents the feasible area; the black grid represents the obstacles; the red circular object and the black circular object represents the search and rescue robot search target and the search and rescue robot. The platform used in the experiments is built on top of a Win10 system with python 3.9.4 installed. The learning factor *α* of Q-learning determines the range of the latest received data that will cover the previous data. When *α*→0, the SAR robot will not learn any information, while when *α*→1, the SAR robot will only read the most recently received information. The discount factor *γ* is another parameter considered in Q-learning learning to determine the type of reward received by the SAR robot. When *γ* = 0, the SAR robot considers only current rewards, and when *γ*→1, the SAR robot considers future rewards. A high discount factor *γ* in range 0≤*γ*≤1 and a low learning rate value *α* in range 0<*α*<1 is a valid combination for Q-learning. Using a high value of the discount factor allows more exploration of Q-learning and prevents Q-learning from falling into local optima, but may lead to instability of the SAR robot during learning. This problem can be improved by using a smaller *α*. Fig 7 shows the influence of different value of *α* on the number of iterations.

To evaluate the generality of the FIQL algorithm applied to SAR robot path planning, we compared FIQL with CQL (classic Q-learning), PSO (Particle Swarm Optimization), GWO (Grey Wolf Optimization), and DA(Dragonfly Algorithm) in different simulation environments. PSO is a calculation to find the global optimal value based on the currently searched optimal value Law. It is not only suitable for single robot planning, but also suitable for path planning of multiple robots; It is easy to fall into local optimum, but its convergence speed is fast. The inspiration of GWO comes from the leadership level and hunting mechanism of gray wolves in nature, with strong convergence performance and few parameters. The inspiration of DA comes from the static and dynamic clustering behavior of dragonflies in nature, which has the advantages of strong stability, fast search speed. The parameter settings of the compared algorithms are shown in Table 1.

### Performance indicators

#### Path length

Path length is one of the important indicators to evaluate the performance of the algorithm. The shorter the path length required for the SAR robot to complete the path planning, the shorter the energy and time consumption is reduced. The distance calculation method we use is the Manhattan distance. The distance between the SAR robot and its search target is calculated as follows:

$$\overset{n}{\underset{i=0}{\sum }}\sqrt{{\left(x_{d}(i+1)-x_{d}(i)\right)}^{2}+{\left(y_{d}(i+1)-y_{d}(i)\right)}^{2}}$$ (8)

where, *i* = 0,1,2…,*n* and *i* = 0 is the host apparatus at the starting position and *i* = *n* is that the pet apparatus meets the search and rescue robot search target. $\left(x_{d}(i),y_{d}(i)\right)$ indicates the current position state of the SAR robot, $\left(x_{d}(i+1),y_{d}(i+1)\right)$ was the next position state of the SAR robot.

#### Convergence time and exploration success rate

Computing time is consumed by the algorithm through iterative computation is denoted as Time(s). The average value of 30 repeated tests in this paper is taken as the computing time. The shorter computing time, the less waiting time for MR to execute the action. The exploration success rate is the number of successful exploration divided by the total number of exploration. The shorter the path length, the shorter the time consumption, and the higher the exploration success rate, the stronger the feasibility of the algorithm.

### Typical grid map path planning for SAR robot

**Fig 8:**
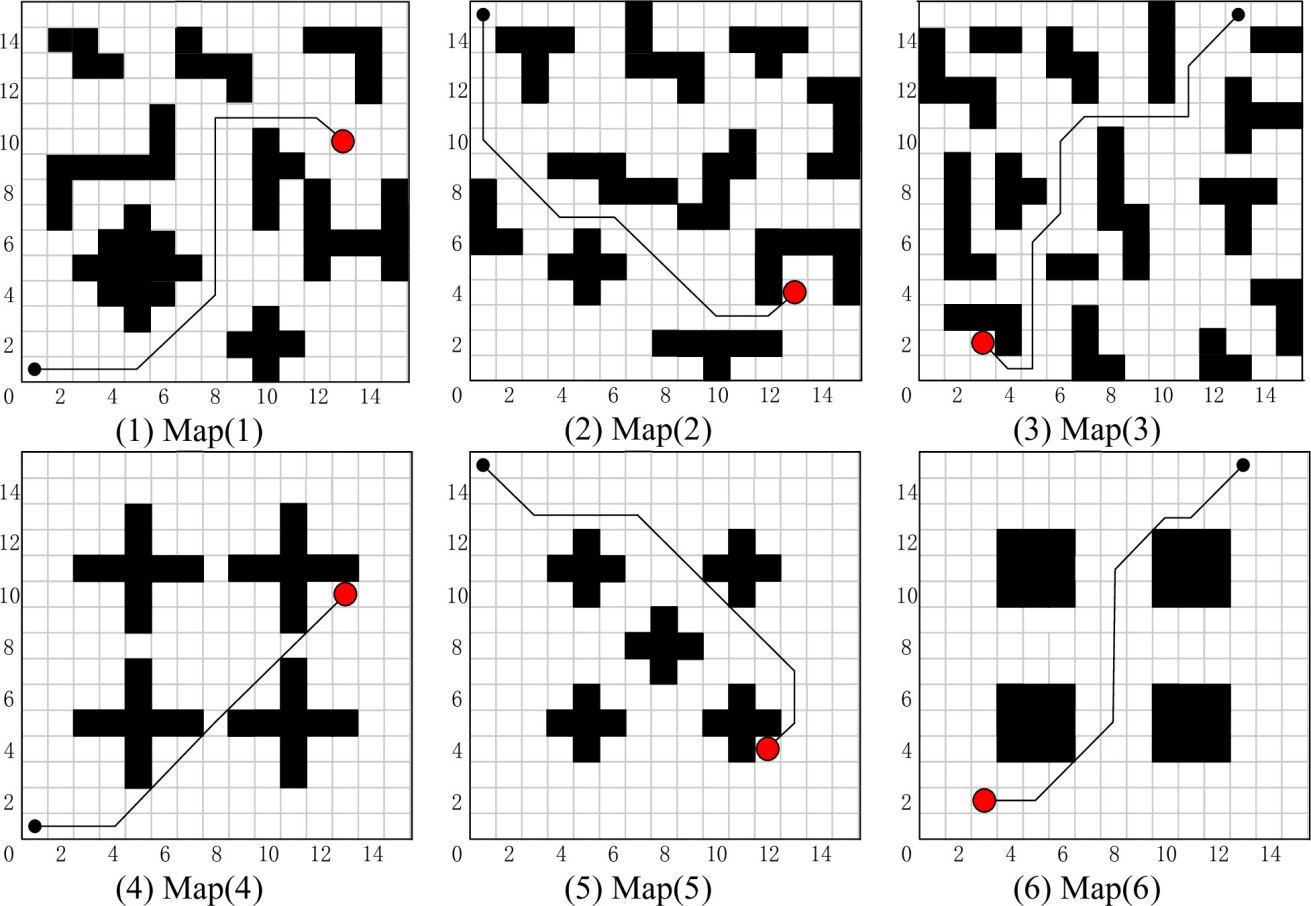
Typical grid map path planning for SAR robot in different environments.

In this section, path planning for SAR using the FIQL in typical grid map is proposed. The proposed algorithm enables the robot to perform the task with the shortest or shorter path length from the starting point to the target point. To verify the effectiveness of the proposed algorithm, tests were conducted on the six grip maps in Fig 8. Each map shows the best path obtained by the FIQL algorithm, the black circle is the starting point and the red circle is the target point.

**Fig 9:**
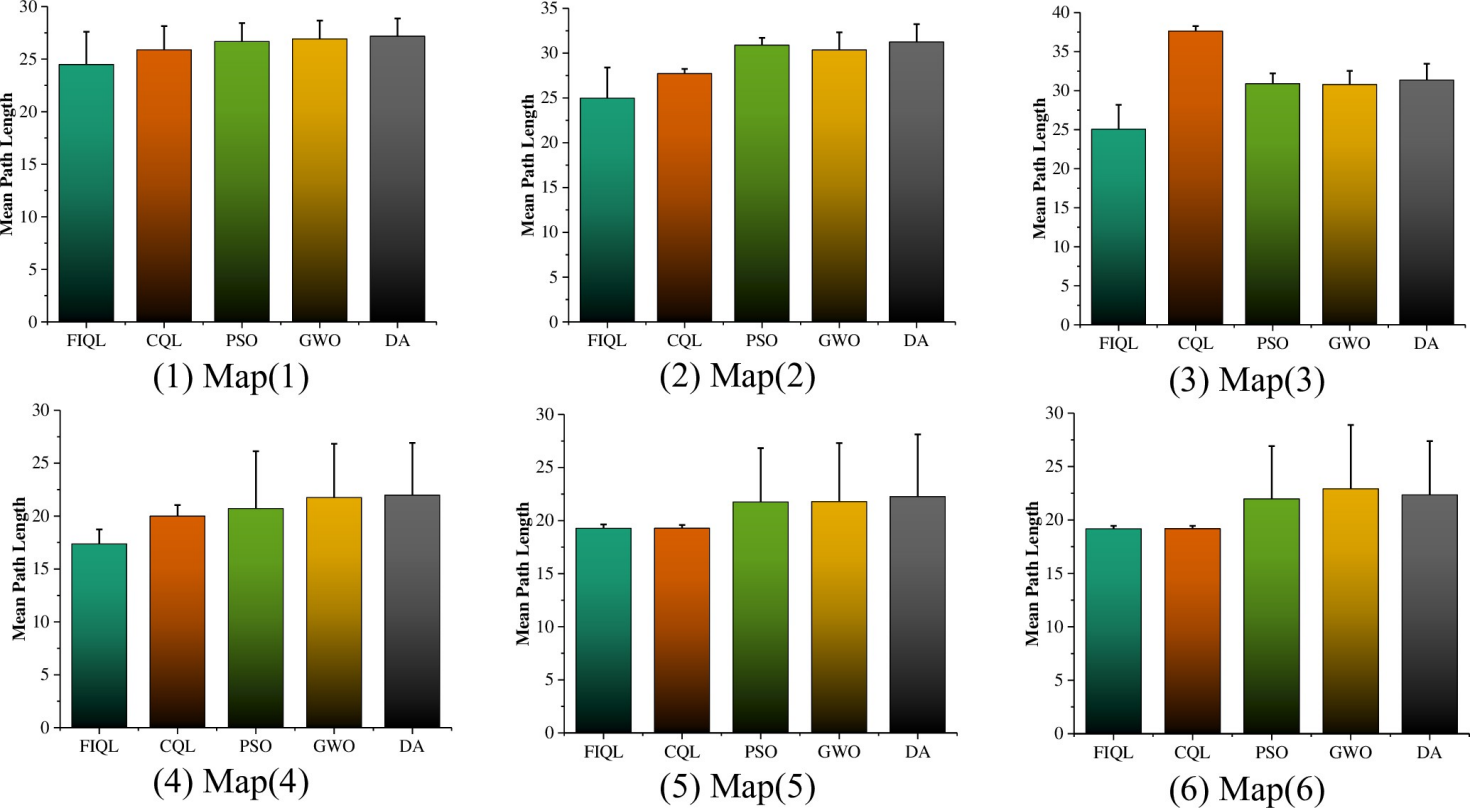
Mean path length of different algorithms in typical grid map for SAR robot.

The optimal results of FIQL in 30 repeated tests are shown in Fig 8. The experimental data and analysis results are recorded in Table 2. The Mean and Std Dev are the average and standard deviation of 30 repeated experiments. The t-test is used to observe whether there is significant difference between the FIQL algorithm and other comparison algorithms. The average path length comparison of various path planning algorithms in typical grid map is shown in Fig 9.

**Table 2:** Comparison between FIQL, CQL, PSO, GWO and DA in typical grid map.

Map	Statistics		FIQL	CQL	PSO	GWO	DA
Map(1)	Path length	Mean	24.489	25.886	26.671	26.911	27.178
		Std Dev.	3.103	2.254	1.750	1.754	1.693
		*t*-test	-	5.07e-2	1.36e-3	4.49e-4	1.04e-4
	Time	Mean	12.045	14.416	10.238	9.872	9.721
Map(2)	Path length	Mean	24.977	27.728	30.892	30.358	31.252
		Std Dev.	3.418	0.517	0.822	1.966	1.984
		*t*-test	-	5.432e-5	1.366e-5	5.241e-10	4.239e-12
	Time	Mean	12.098	14.670	10.205	10.071	10.138
Map(3)	Path length	Mean	25.045	27.613	30.892	30.778	31.353
		Std Dev.	3.142	0.659	1.320	1.751	2.082
		*t*-test	-	4.998e-5	2.973e-13	3.721e-12	7.147e-13
	Time	Mean	12.097	14.758	10.176	10.167	10.134
Map(4)	Path length	Mean	17.365	20.001	20.711	21.750	21.971
		Std Dev.	1.372	1.041	5.421	5.078	4.947
		*t*-test	-	1.337e-11	3.551e-13	3.241e-4	2.689e-16
	Time	Mean	11.043	13.152	9.762	9.773	9.763
Map(5)	Path length	Mean	19.279	21.281	21.751	21.786	22.251
		Std Dev.	0.366	0.309	5.078	5.522	5.878
		*t*-test	-	9.771e-1	1.012e-2	1.161e-2	7.636e-3
	Time	Mean	11.195	13.196	9.159	8.944	8.959
Map(6)	Path length	Mean	19.170	21.184	21.971	22.915	22.349
		Std Dev.	0.283	0.271	4.947	5.981	5.034
		*t*-test	-	8.455e-1	3.026e-3	1.135e-3	1.043e-3
	Time	Mean	11.223	13.465	9.180	8.983	8.917

### Improved grid map for SAR robot

In this section, path planning for SAR using the FIQL in improved grid map is proposed. In order to verify the impact of improved grid map on route selection and success rate of path planning, the grid environment of SAR in this section is the same as that in the previous section. They all start from the same location and the target point location is the same.

**Fig 10:**
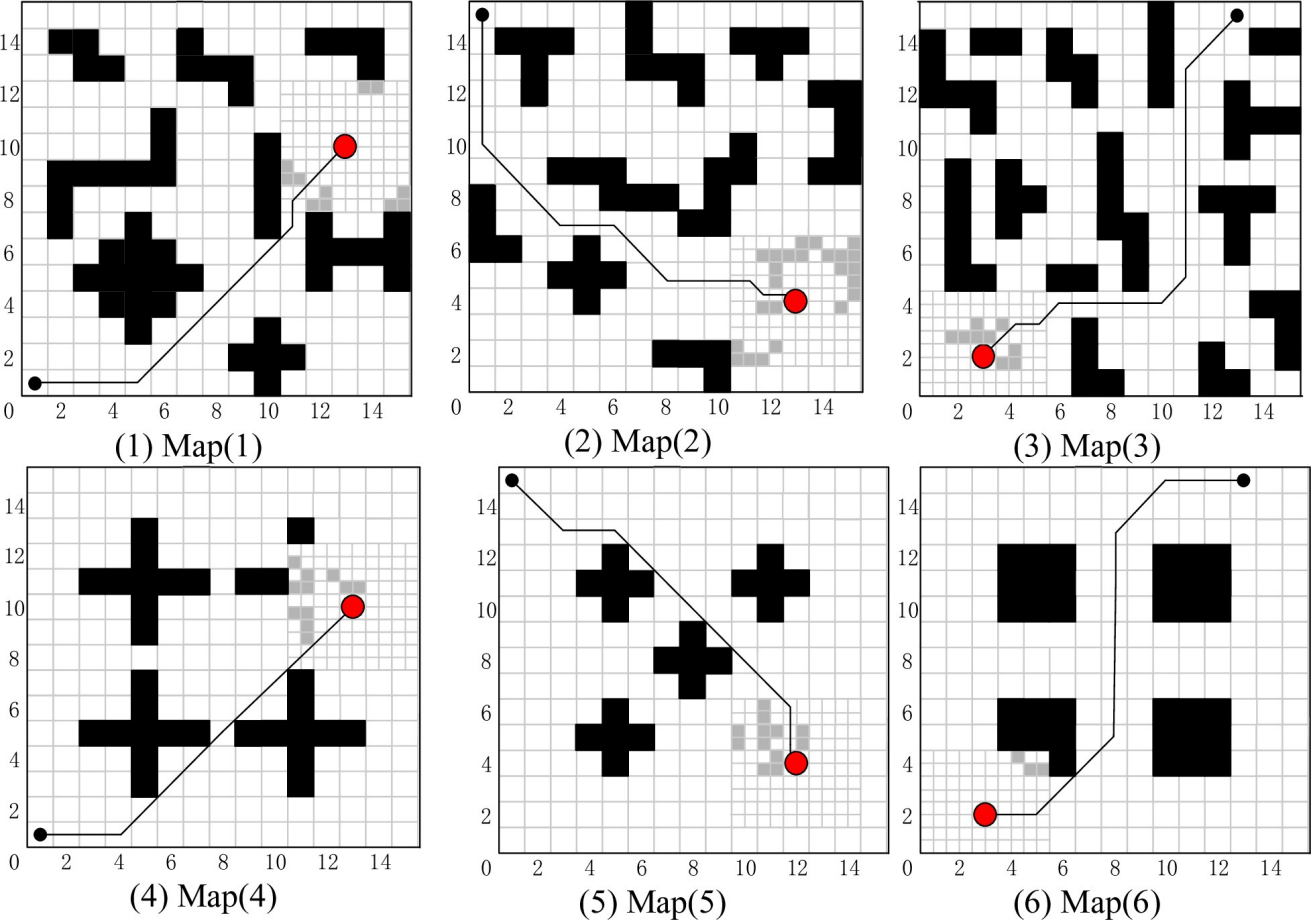
The optimal path in the improvement gird map using FIQL algorithm.

The optimal results of FIQL in 30 repeated tests are shown in Fig 10. The paths of other compared algorithms are not indicated in Fig 10 for a clearer representation of the paths of FIQL.

**Fig 11:**
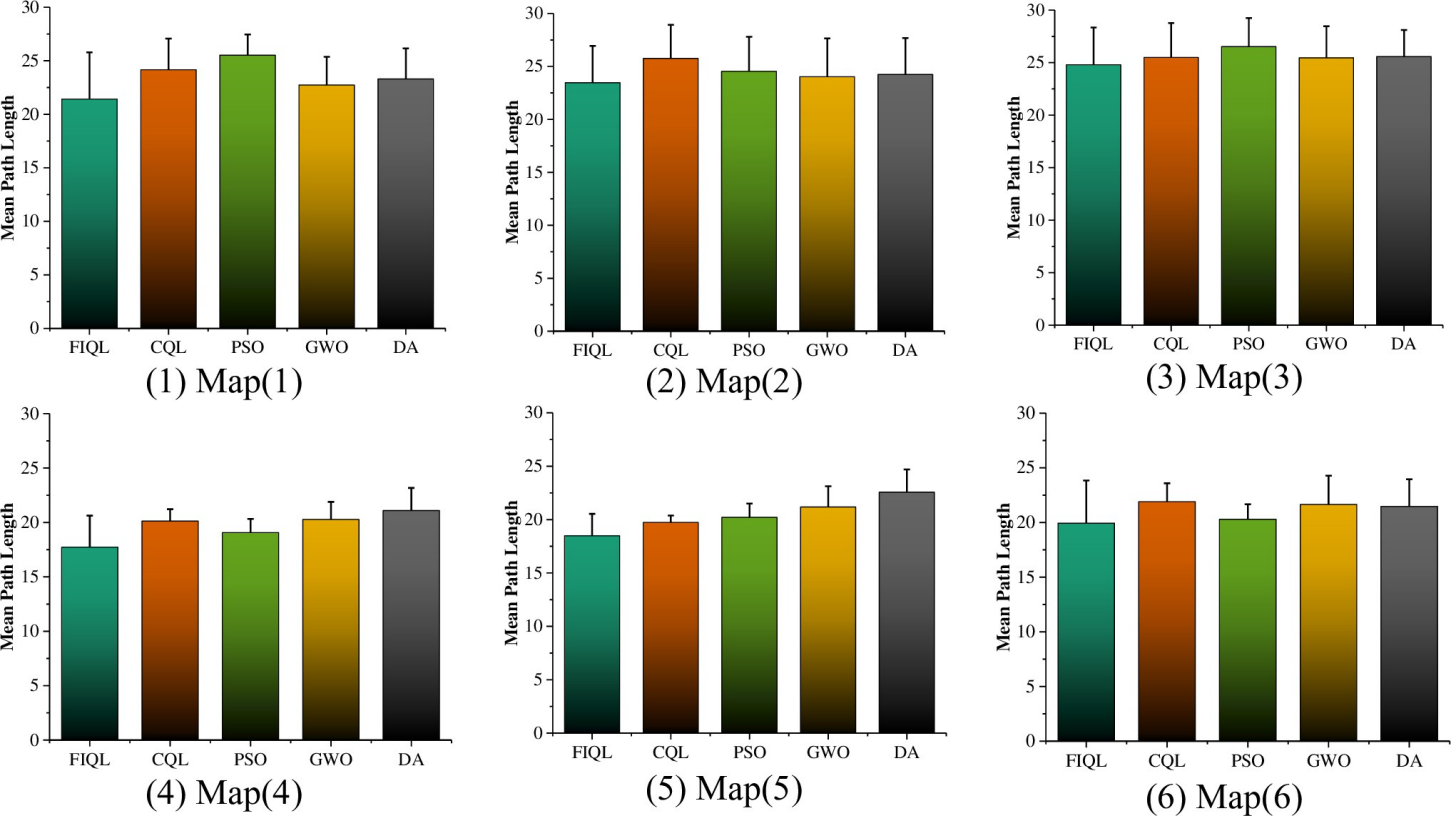
Mean path length of different algorithms in improved grid map for SAR robot.

It can be seen that in the six test grid maps, FIQL can effectively solve the path planning problem of SAR robot from the starting position to the target position without collision with obstacles. The results in Tables 2 and 3 show that FIQL can effectively reduce the random motion in the initial stage of SAR robot, further accelerate the convergence speed and reduce the calculation time. The average path length comparison of various path planning algorithms in improved grid map is shown in Fig 11. Compared with CQL, FIQL can achieve the optimal path length in different environments, which proves the practicability, generality, and robustness of this method. However, through the statistical results, we can find the limitations of FIQL: the calculation time is long, but it is within the acceptable range. To verify that FIQL is superior to CQL in path length, calculation time, and exploration success rate. The improvement percentage of CQL in this paper is recorded in Tables 4 and 5. Table 4 shows the statistical results of SAR robot in the typical grid map, and Table 5 shows the statistical results of SAR robot in the improved grid map.

**Table 3:** Comparison between FIQL, CQL, PSO, GWO and DA in improved grid map.

Map	Statistics		FIQL	CQL	PSO	GWO	DA
Map(1)	Path length	Mean	21.421	24.151	25.591	22.728	23.299
		Std Dev.	4.368	2.912	1.938	2.638	2.851
		*t*-test	-	6.072e-3	1.661e-5	1.658e-5	5.324e-2
	Time	Mean	11.184	13.838	9.795	10.201	8.872
Map(2)	Path length	Mean	23.465	25.759	24.534	24.204	24.238
		Std Dev.	3.483	3.167	3.275	3.627	3.446
		*t*-test	-	9.845e-3	2.261e-1	4.244e-1	3.909e-1
	Time	Mean	11.551	13.364	11.264	9.772	10.064
Map(3)	Path length	Mean	24.801	25.493	26.524	25.456	25.571
		Std Dev.	3.543	3.271	2.727	3.001	2.535
		*t*-test	-	4.434e-1	3.899e-2	4.423e-1	3.362e-1
	Time	Mean	12.097	14.744	10.223	10.764	11.846
Map(4)	Path length	Mean	17.718	20.128	19.061	20.272	21.094
		Std Dev.	2.904	1.087	1.256	1.619	2.077
		*t*-test	-	7.674e-5	2.362e-2	7.089e-5	2.928e-6
	Time	Mean	10.571	11.692	8.842	8.757	9.035
Map(5)	Path length	Mean	18.475	19.737	20.207	21.173	22.569
		Std Dev.	2.062	0.647	1.301	1.957	2.132
		*t*-test	-	1.136e-2	1.688e-3	2.623e-5	9.594e-9
	Time	Mean	11.756	12.784	8.826	8.433	8.686
Map(6)	Path length	Mean	19.929	21.895	20.291	21.647	21.459
		Std Dev.	3.908	1.688	1.381	2.628	2.489
		*t*-test	-	4.254e-2	6.991e-1	6.658e-3	5.023e-2
	Time	Mean	10.912	11.916	8.207	8.822	9.956

The experimental data and analysis results are recorded in Table 3. The Mean and Std Dev are the average and standard deviation of 30 repeated experiments. The t-test is used to observe whether there is significant difference between the FIQL algorithm and other comparison algorithms.

**Table 4:** Comparison of performance between FIQL and CQL in typical grid map.

	Path Length	Time	Success Rate
	VS CQL	VS CQL	VS CQL
Map(1)	5.39%	16.45%	6.67%
Map(2)	9.92%	17.53%	6.66%
Map(3)	9.29%	18.03%	6.67%
Map(4)	13.17%	16.03%	6.70%
Map(5)	9.41%	15.16%	6.67%
Map(6)	9.51%	16.65%	3.33%

**Table 5:** Comparison of performance between FIQL and CQL in improved grid map.

	Path Length	Time	Success Rate
	VS CQL	VS CQL	VS CQL
Map(1)	11.30%	19.17%	6.66%
Map(2)	8.91%	13.56%	6.67%
Map(3)	2.71%	17.95%	6.67%
Map(4)	11.97%	9.59%	6.66%
Map(5)	6.39%	8.04%	6.66%
Map(6)	8.97%	8.43%	6.66%

### Analysis of simulation experiment results

In Figs 8 and 10, we can find that the paths obtained in the improved grid map and typical grid map using the FIQL algorithm are different. The statistical results in Tables 4 and 5 indicates the accuracy of the modeling in the improved grid map is improved compared with the classical grid map, and the SAR robot can pass through those areas that cannot pass in the classical grid map. The exploration accuracy and the success rate of exploration are also improved to different degrees. We can also find the path lengths obtained in the improved grid map are shorter than those before the improvement. In Tables 4 and 5, it can be found that the success rate of FIQL exploration before the map improvement is higher than CQL.

Map (1)—Map (3) in Figs 8 and 10, we designed a path planning task in a dense and irregular environment. In these three maps, we designed a local optimum to simulate the path planning problem under dense obstacles that may be encountered in the real environment. Simulation results show that FIQL is robust and universal. Map (4)—Map (6) in Figs 8 and 10, we design the path planning task in the rule environment. The experimental results show that the FIQL algorithm can make the mobile robot plan the shortest path. The suboptimal algorithm is GWO algorithm, which performs well in most experiments, but is not as stable as FIQL algorithm. During the experiment, CQL could not find the global optimal solution due to its slow convergence speed, resulting in a long tortuous path length. Overall, FIQL, CQL, PSO, GWO and DA are able to perform the path planning task, but the performance results are differ, FIQL has shortest path length, but calculation time is longer than PSO, GWO and DA.

## Conclusions

In this paper, a SAR robot search algorithm based on FIQL algorithm is proposed and simulation experiments are conducted. Compared with the comparison algorithm, the initialization of the Q-table by FPA reduces the action selection blindness of the SAR robot, and the convergence time is shorter and the length of the explored path is more in line with the real-life needs. In the environment modeling part, the grid where the obstacles are located is modeled as static grid and follow-me grid according to the location distance from the search target of the SAR robot, which greatly increases the accuracy of the environment modeling, reduces the possibility of collision of the SAR robot, and increases the probability of successful exploration of the SAR robot. Based on the static and dynamic settings of the reward function, different feedback values are obtained by interacting with the environmental information in real time under different circumstances to select the direction of action at different moments, which can finally achieve the guidance of the SAR robot to the search and SAR robot search target location.

The results show that the improved grid map can increase the exploration success rate of the algorithm. FIQL can better complete the path planning task in different grid maps and has the shortest path length than CQL, PSO, GWO and DA.
